# Impact of Vitamin D3 Supplementation on 28-Day ICU Mortality in Sepsis Patients: A Retrospective Study with Propensity Score Matching

**DOI:** 10.3390/pathogens15040433

**Published:** 2026-04-16

**Authors:** Xiaofei Huang, Anqiang Zhang, Dalin Wen, He Li, Ling Zeng

**Affiliations:** 1 Department of Emergency Surgery, The Second Affiliated Hospital of Anhui Medical University, Hefei 230601, China; 2 Department of Emergency, Affiliated Hospital of Zunyi Medical University, Zunyi 563003, China; 3 Department of Trauma Medical Center, State Key Laboratory of Trauma and Chemical Poisoning, Daping Hospital, Army Medical University, Chongqing 400042, China

**Keywords:** vitamin D3, intensive care, sepsis, mortality

## Abstract

Reduced levels of vitamin D are associated with increased incidence and mortality of sepsis. Nonetheless, the effectiveness of vitamin D supplementation in improving sepsis patients’ outcomes continues to be debated. In this research, which was conducted as a retrospective cohort analysis, data obtained from the Medical Information Mart for Intensive Care IV (MIMIC-IV 3.0) were used. The focus of the study was on vitamin D3 administration to sepsis patients while in the ICU. The primary outcome measurement was 28-day ICU mortality, with secondary outcomes of mechanical ventilation duration, percentage of patients receiving mechanical ventilation, and ICU stay length. The Kaplan–Meier curve analysis, Cox regression analysis, and subgroup analyses were performed to explore the link between vitamin D3 supplementation and sepsis prognosis. A 1:1 propensity score matching (PSM) approach was used to strengthen the reliability of the results. Before matching, the cohort comprised 28,524 patients, which was reduced to 4,856 after PSM. The analysis revealed that vitamin D3 supplementation was associated with a lower 28-day ICU mortality rate (HR = 0.71, 95% CI: 0.64–0.78, *p* < 0.001). Kaplan–Meier curve analysis revealed significantly greater survival probabilities in the group receiving vitamin D3 than in the group not receiving vitamin D3 (*p* < 0.001). Subgroup analysis showed that total cumulative exposure to vitamin D3 was more strongly associated with 28-day ICU mortality (*p* < 0.001), whereas daily dose and dosing frequency showed no significant association. The results after PSM and subgroup analysis were consistent with those of the original cohort study, further confirming the robustness of the results. Overall, vitamin D3 supplementation is associated with lower 28-day ICU mortality and better outcomes in patients with sepsis. However, given the retrospective observational design, large-scale prospective randomized controlled trials are warranted to validate these observational associations and establish causal effects.

## 1. Introduction

Given the high rates of illness and death linked to sepsis [1,2,3], the World Health Organization (WHO) has identified sepsis as a critical global health issue. The defining feature of sepsis is a dysregulated immune response of the host to infection [4]. This immune dysregulation might persist throughout the entire course of the disease and even continue for weeks or months after recovery [5]. Immune dysregulation prevents the clearance of the primary infection and increases the risk of subsequent secondary/opportunistic infections, which contributes to poor outcomes for sepsis patients [5]. Therefore, identifying immune modulation strategies, alongside antibiotic therapy, resuscitation, and organ support, plays a vital role in improving outcomes for sepsis patients.

Traditionally, vitamin D functions primarily to regulate calcium and phosphate metabolism. The active form, 1,25-dihydroxyvitamin D, is crucial for both sustaining healthy mineralized bones and functioning as an immune regulator [6,7]. Immunoregulation includes stimulating various immune cells, such as T lymphocytes, B lymphocytes, macrophages, and dendritic cells, and increasing the generation of antimicrobial peptides while improving neutralization ability [8,9,10]. Vitamin D deficiency leads to immune system dysfunction, which might trigger or exacerbate autoimmune diseases, including type 1 diabetes, autoimmune adrenal diseases, multiple sclerosis [11], and Hashimoto’s thyroiditis [8,12,13], and increase susceptibility to bacterial and viral infections [7]. Multiple studies have demonstrated that reduced serum 25(OH)D levels correlate with higher incidence of sepsis, higher mortality rates, and extended ICU stays among sepsis and critically ill patients [14,15,16,17,18]. Vitamin D supplementation might have positive preventive effects on sepsis occurrence [19] and the prognosis of acute respiratory failure and sepsis-associated acute kidney injury [20,21]. Although findings from a recent study indicated that vitamin D treatment might decrease the mortality rate in patients with sepsis [22], its clinical efficacy is still a matter of debate. Consequently, we employed extensive clinical data to clarify the connection between vitamin D3 therapy and sepsis patient outcomes. This study suggests that vitamin D3 supplementation is associated with better outcomes in sepsis patients, generating the hypothesis of a potential beneficial effect that warrants confirmation in future randomized controlled trials.

## 2. Method

### 2.1. Data Source

**Figure 1:**
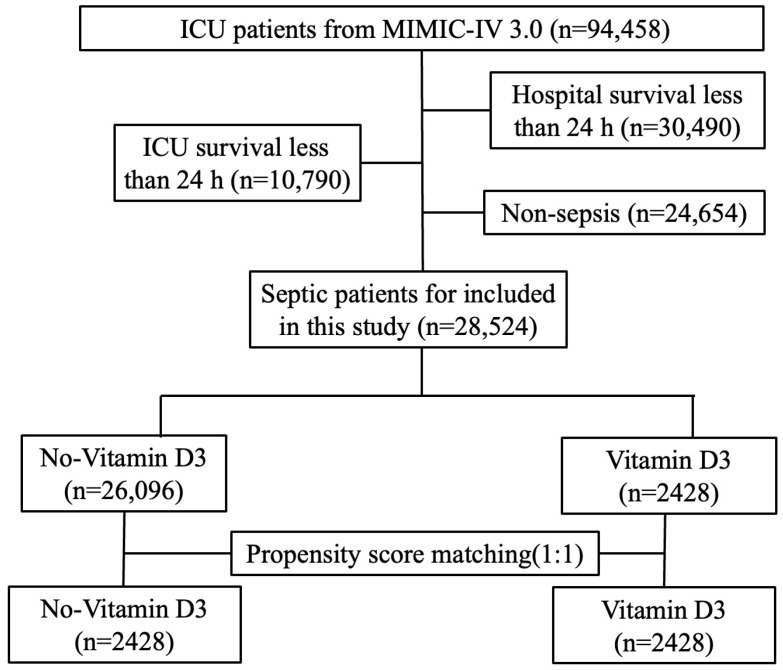
Flowchart of patient selection. After excluding patients with ICU or hospital stay less than 24 h and non-septic patients, 28,524 septic patients were included. Following 1:1 propensity score matching, 2428 patients receiving Vitamin D3 treatment were matched with 2428 controls without Vitamin D3.

In this study, version 3.0 of the Medical Information Mart for Intensive Care IV (MIMIC-IV) database was utilized. The MIMIC-IV 3.0 database contains the data of over 90,000 patients from the intensive care unit (ICU) of the Beth Israel Deaconess Medical Center, Boston, Massachusetts, between 2008 and 2022. The database is normatively administered and contains high-quality data on demographics, laboratory tests, vital signs, disease diagnoses and survival status. In total, 94,458 patients with first-time cases of sepsis treated in the ICU were included in the MIMIC-IV 3.0 database. Patients were excluded if they met the following criteria: (1) were younger than 18 years; (2) had an ICU stay shorter than 24 h; and (3) were not diagnosed with sepsis. The relevant data were subsequently obtained by one of the authors (Xiaofei Huang), and the author obtained the necessary certification (certification number: 59248579). Informed consent from individuals was not needed, as patient privacy in this database is maintained through the anonymization of data. The flowchart of patient selection is shown in Figure 1.

### 2.2. Data Extraction

Data gathered during the first 24 h following ICU admission for each patient were retrieved from the MIMIC-IV 3.0 database. The demographic data, vital signs, laboratory test results, clinical scores, and medical history were extracted, and vitamin D3 therapy was provided during the ICU stay. In addition, comorbidity data were extracted by identifying the related International Classification of Diseases codes. Variables with less than 20% missing data were retained and imputed using multiple imputation methods.

### 2.3. Exposure and Outcomes

The exposure was vitamin D3 supplementation during the ICU stay, with no restrictions. Vitamin D3 was administered via enteral routes (oral, nasogastric tube, gastrostomy tube) and parenteral routes (intravenous injection). Data regarding vitamin D3 exposure were extracted from the prescription tables. Patients who had missing data regarding vitamin D3 supplementation exposure were not included in the analysis. The primary endpoint was 28-day ICU mortality. The secondary endpoints included the duration of mechanical ventilation, the percentage of patients receiving mechanical ventilation, and the length of ICU stay.

### 2.4. Statistical Analysis

Continuous data were presented as medians with interquartile ranges (IQRs), and group differences were analyzed using the Mann–Whitney U test. Categorical variables were presented as frequencies or percentages and were assessed with the chi-square test. The association between vitamin D3 supplementation and mortality in sepsis patients was evaluated via a Cox regression model. Kaplan–Meier (KM) curves were analyzed to assess the impact of vitamin D3 on survival. Subgroup analyses were also performed to explore the effect of vitamin D3 supplementation on 28-day mortality, considering variables such as age, sex, hypertension, diabetes, chronic obstructive pulmonary disease (COPD), and malignancies. All the statistical analyses were carried out with R software (R version 4.2.2, Austria), along with Zstats v0.90 www.medsta.cn/software (accessed on 15 November 2025) and MSTATA software v0.92 www.mstata.com/ (accessed on 23 November 2025).

### 2.5. Propensity Score Matching

To minimize confounding bias caused by differences in baseline characteristics between the vitamin D3 supplementation group and the non-supplementation group, 1:1 propensity score matching (PSM) was performed to balance the baseline characteristics of the two groups. Propensity scores were calculated using a multivariable logistic regression model. Covariates included potential confounders that may affect the use of vitamin D3 and the prognosis of sepsis, such as age, sex, hypertension, diabetes, COPD, and malignancies. The balance of baseline characteristics between the two groups was assessed using standardized mean differences (SMD), with an SMD < 0.1 considered indicative of adequate balance. The original cohort included 28,524 patients before matching. After 1:1 PSM, 2428 patients were included in each group, which improved the reliability and comparability of the study results.

### 2.6. Subgroup Analyses

To investigate the association between vitamin D3 supplementation and 28-day ICU mortality in patients with sepsis, we performed subgroup analyses in the pre-matching and post-matching cohorts according to age (<65 years, ≥65 years), sex, hypertension, diabetes, COPD, malignancy, serum vitamin D level, and year of admission. Serum vitamin D levels were classified based on the definition used in the MIMIC IV database: deficient (<30 ng/mL), normal (30–60 ng/mL), and sufficient (>60 ng/mL). However, due to the limited number of patients in the sufficient group (*n* = 5), subgroup analysis was performed only for the deficient and normal groups. Based on the key updates of the Surviving Sepsis Campaign guidelines in 2012 and 2016 [23,24,25,26], patients were stratified by admission year into early, middle, and recent periods.

To further investigate the effect of vitamin D3 dosing regimens on study outcomes, we performed additional stratified analyses among treated patients, with stratification criteria based on prior literature [27] and actual vitamin D3 administration records from the MIMIC-IV 3.0 database. Specifically, patients were stratified into three subgroups: by cumulative dose (low cumulative dose ≤ 25,000 IU vs. high cumulative dose > 25,000 IU), by daily dose (low daily dose ≤ 2000 IU/day vs. high daily dose > 2000 IU/day), and by dosing frequency (infrequent dosing 1/day vs. frequent dosing > 1/day). All analyses were adjusted for age, sex, mechanical ventilation status, diabetes mellitus, SOFA (Sequential Organ Failure Assessment) score, and Charlson comorbidity index.

## 3. Results

### 3.1. Patient Selection

The patient selection process is illustrated in Figure 1. From the MIMIC-IV database, 94,458 patients were initially selected. After 30,490 patients whose hospital stay was less than 24 h and 10,790 patients whose ICU stay was less than 24 h were excluded, 53,178 patients remained. Subsequently, 24,654 nonseptic patients were excluded. Finally, a cohort of 28,524 patients was considered for this study. Among these patients, 2428 received vitamin D3 treatment during their ICU stay. After 1:1 propensity score matching, 4856 patients were included (2428 in each group).

### 3.2. Cohort Characteristics

The baseline demographic characteristics, vital signs, laboratory indicators, and comorbidity details are presented in Table 1. In the pre-matched cohort, the median age of patients receiving vitamin D3 treatment was 71 years (62–81), with a median body weight of 77.53 kg (64.70–93.40), and 51.57% were male. Compared with patients who did not receive vitamin D3, those who received vitamin D3 were older, had a higher body weight, and included a higher percentage of males. Additionally, patients treated with vitamin D3 presented with lower heart rates (HR); white blood cell (WBC) counts; red blood cell (RBC) counts; and hemoglobin, sodium, chloride, and lactate levels. In contrast, patients treated with vitamin D3 showed higher red cell distribution width (RDW), total calcium, prothrombin time (PT), international normalized ratio (INR), and creatinine levels than those not treated with vitamin D3. Moreover, the incidence of comorbidities such as hypertension, diabetes, myocardial infarction, and COPD was lower in the treated group. After PSM, all SMDs for the covariates were less than 0.1, with no notable differences in baseline characteristics observed between the groups, confirming adequate balance post-matching. The distributions of the SMD and probability density after PSM matching are presented in Supplemental Figures S1 and S2.

### 3.3. Clinical Outcomes

**Figure 2:**
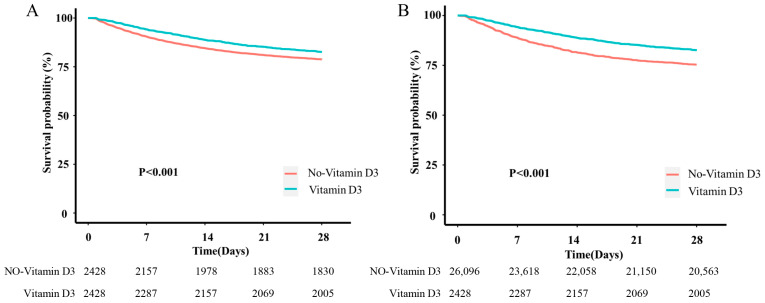
Survival probability trends for patients who received and did not receive vitamin D3 treatment. (**A**) Survival probability trends in the original cohort before PSM. (**B**) Survival probability trends after PSM. Patients receiving Vitamin D3 exhibited significantly improved survival compared to matched controls.

As shown in Table 2, patients receiving vitamin D3 had a lower 28-day ICU mortality rate (17.42%, 423/2428) than those not receiving vitamin D3 (21.20%, 5533/26,096). After 1:1 PSM, both cohorts comprised 2428 patients. The 28-day ICU mortality rate was 17.42% (423/2428) in the vitamin D3 group and 24.63% (598/2428) in the non-vitamin D3 group. Univariate Cox proportional hazards regression analysis revealed that the hazard ratio (HR) for vitamin D3 therapy was 0.79 (95% CI: 0.72–0.87, *p* < 0.001) before matching and 0.66 (95% CI: 0.59–0.75, *p* < 0.001) after matching, suggesting that vitamin D3 therapy was associated with lower 28-day ICU mortality among sepsis patients. The multivariate Cox proportional hazards regression analysis yielded consistent results, with an HR of 0.71 (95% CI: 0.64–0.78, *p* < 0.001) before matching and 0.66 (95% CI: 0.58–0.75, *p* < 0.001) after matching (Table 3). The Kaplan–Meier curves for 28-day ICU mortality in the pre- and post-matching cohorts on the basis of vitamin D3 usage are shown in Figure 2A,B, respectively. The results indicated that vitamin D3 treatment was associated with significantly higher survival rates in patients with sepsis (*p* < 0.001).

For the secondary outcomes, a lower percentage of patients receiving mechanical ventilation was observed in the vitamin D3 group (84.1% vs. 87.7%, *p* < 0.001). Moreover, the median duration of mechanical ventilation was 37.0 (IQR, 11.6–92.3) hours, which was significantly shorter than that of patients who did not receive vitamin D3 (41.0 (IQR, 16.8–96.0) (*p* = 0.002)). In the PSM cohort, similar results were observed for the duration (37.0 (IQR, 11.6–92.3) vs. 42.0 (IQR, 17.3–93.8); *p* = 0.012) and percentage of patients receiving (84.1% vs. 87.7%, *p* < 0.001) mechanical ventilation. However, the ICU length of stay was not significantly different between the groups before and after matching (Table 2).

### 3.4. Vitamin D3 Regimen

To further investigate the effect of vitamin D3 dosing regimens on study outcomes, we conducted stratified analyses among patients who received treatment based on the cumulative dose, daily dose, and dosing frequency. Results indicated that, when stratified by cumulative dose, both the unadjusted analysis (HR = 0.41, 95% CI: 0.27–0.64, *p* < 0.001) and the adjusted analysis (HR = 0.37, 95% CI: 0.24–0.58, *p* < 0.001) indicated that the high-dose group was significantly associated with a reduced risk of 28-day ICU mortality. By contrast, stratified analyses according to daily dose or dosing frequency revealed no significant differences in mortality risk between different levels within each indicator, regardless of adjustment. In the daily dose analysis, the unadjusted HR was 0.77 (95% CI: 0.40–1.49, *p* = 0.441) and the adjusted HR was 0.68 (95% CI: 0.35–1.32, *p* = 0.257). In the dosing frequency analysis, the unadjusted HR was 0.80 (95% CI: 0.41–1.56, *p* = 0.516) and the adjusted HR was 0.77 (95% CI: 0.40–1.50, *p* = 0.444). These results indicated that, within the study cohort, the total cumulative exposure to vitamin D3 showed a more pronounced association with clinical outcomes, whereas daily dose and dosing frequency were not significantly associated with outcomes (Table 4).

### 3.5. Subgroup Analysis

**Figure 3:**
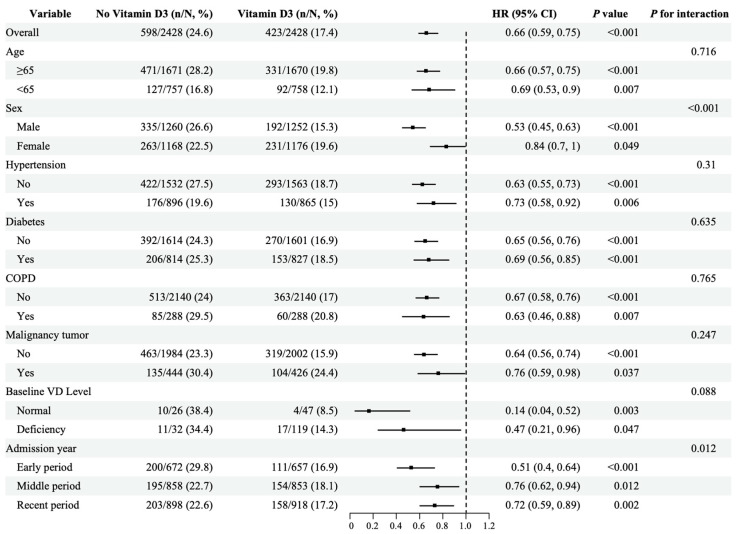
Subgroup analysis results after propensity score matching. The vertical dashed line represents the reference line at a HR of 1.0, indicating no difference in risk between groups. The protective association of vitamin D3 with lower 28-day ICU mortality persisted across nearly all subgroups, with statistically significant interactions observed for sex and admission year.

To explore the association between vitamin D3 administration and 28-day ICU mortality in sepsis patients, we conducted subgroup analyses stratified by age (<65 years, ≥65 years), sex, hypertension, diabetes, COPD, malignancy, baseline serum 25-hydroxyvitamin D level, and year of admission. As shown in Figure 3, statistically significant interactions were observed only in the sex subgroup (*p* < 0.001) and the admission year subgroup (*p* = 0.012), while no significant interactions were detected in the other subgroups after PSM. In the stratification by baseline vitamin D level, vitamin D3 supplementation was associated with reduced mortality in both the normal vitamin D subgroup (HR = 0.14, 95% CI: 0.04–0.52, *p* = 0.003) and the deficient vitamin D subgroup (HR = 0.47, 95% CI: 0.21–0.96, *p* = 0.047), with a borderline significant interaction between the two groups (*p* = 0.088). Furthermore, across all three admission year subgroups, sepsis patients who received vitamin D3 supplementation exhibited significantly lower 28-day ICU mortality than those who did not: early period (HR = 0.51, 95% CI: 0.40–0.64, *p* < 0.001), middle period (HR = 0.76, 95% CI: 0.62–0.94, *p* = 0.012), and recent period (HR = 0.72, 95% CI: 0.59–0.89, *p* = 0.002). Although a significant interaction was observed across different time subgroups (*p* = 0.012), vitamin D3 supplementation was consistently associated with lower 28-day ICU mortality across all time subgroups. These findings suggested that the protective association of vitamin D3 supplementation with lower 28-day ICU mortality among sepsis patients is consistent in direction across various clinical subgroups, supporting the robustness of the main conclusions.

## 4. Discussion

In this study, vitamin D3 supplementation was associated with lower 28-day ICU mortality in critically ill patients with sepsis. Similar associations were observed across subgroup and sensitivity analyses.

Vitamin D is an important immune modulator and is associated with susceptibility to infections. The kidneys are the main site where vitamin D is activated to 1,25(OH)_2_D [28]. In cases of acute kidney injury linked to sepsis, a decline in renal function results in a notable decrease in 1,25(OH)_2_D levels. Administering exogenous vitamin D has been shown to increase the 28-day survival rate of patients with sepsis-induced acute kidney injury [21]. Vitamin D deficiency is present not only in patients with kidney injury but also in the majority of severely ill individuals. Studies have shown that up to 77% of individuals with severe illness suffer from vitamin D deficiency, which might be due to insufficient sunlight exposure and malnutrition, leading to reduced epidermal vitamin D production, as well as the conversion of 25(OH)D to active 1,25(OH)_2_D to meet the increased demand of the body, especially for the immune-modulating effects mediated by 1,25(OH)_2_D [29]. As mentioned earlier, several studies reported that reduced serum 25(OH)D levels are correlated with increased incidence of sepsis, increased mortality rates, and prolonged ICU stays in sepsis patients and critically ill patients. Earlier research has indicated that administering exogenous vitamin D3 does not significantly improve outcomes for ICU patients with sepsis [30]. However, a recent study evaluated the efficacy and safety of small and large doses of vitamin D3 administered enterally to 80 septic patients requiring mechanical ventilation. The results revealed that early high-dose vitamin D3 given enterally reduced serum procalcitonin levels, elevated serum IL-37 levels, and increased disease severity scores [31]. Differences in research findings, apart from patient heterogeneity, might also be attributed to variations in administration methods. An animal study revealed that both oral cholecalciferol and intravenous calcitriol alleviated sepsis-induced acute lung injury in obese mice. However, oral cholecalciferol had a more pronounced effect on regulating the Th/Treg cell balance and was more likely to affect anti-inflammatory pathways associated with the renin–angiotensin system in the lungs [32]. In this study, large-scale clinical data were analyzed, which revealed that the use of vitamin D3 was linked to lower 28-day ICU mortality among patients with severe sepsis, which aligns with findings from Li et al. [33]. Notably, we found that the total cumulative exposure to vitamin D3 was more strongly associated with 28-day ICU mortality (*p* < 0.001), whereas daily dose and dosing frequency showed no significant association. This finding is consistent with previous literature, suggesting that achieving sufficient cumulative exposure, rather than adhering to a specific dosing schedule, may be more relevant to the observed association in ICU patients with sepsis [34,35]. In addition, vitamin D3 supplementation was associated with a lower incidence and shorter duration of mechanical ventilation. Mechanistically, previous studies have indicated that vitamin D may alleviate lung injury by modulating inflammatory responses, preserving the integrity of the pulmonary epithelial barrier, and promoting epithelial repair [36]. Furthermore, our findings showed that the association between vitamin D3 supplementation and lower 28-day ICU mortality appeared more pronounced in female patients than in male patients, suggesting a potential sex-based difference in this observed association. The potential biological basis of this sex difference remained incompletely understood, but accumulating evidence indicated that sex hormones, especially estrogen, modulated vitamin D metabolism and signaling pathways in a sex-dependent manner. The biologically active form of vitamin D, 1α,25-dihydroxyvitamin D_3_ [1,25-(OH)_2_D_3_], exerted its effects mainly by binding to the vitamin D receptor (VDR) and initiated downstream transcriptional regulation. Prior studies indicated that estrogen may augment vitamin D signaling and increased its availability through dual regulatory mechanisms: on the one hand, estrogen upregulated VDR expression in various tissues of humans and rats, and enhanced cellular responsiveness to 1,25-(OH)_2_D_3_ [37,38,39]. In addition, CYP24A1—the cytochrome P450 subunit of 25-hydroxyvitamin D_3_-24-hydroxylase—was a key enzyme mediating vitamin D degradation and clearance. Estrogen had been shown to suppress CYP24A1 expression, thereby reducing vitamin D catabolism [40,41,42]. Therefore, future prospective, multicenter studies should be conducted to increase the accuracy of the findings. In the study design, the dosage, administration route, and duration of vitamin D3 treatment should be clearly defined, and participants’ serum 25(OH)D levels should be regularly monitored to ensure that these levels remain within the effective range.

The protective association of vitamin D3 supplementation with lower 28-day ICU mortality in sepsis patients may be mediated by several mechanisms: First, the immune-modulating effects of vitamin D might play a key role. Animal studies have suggested that vitamin D regulates the Th/Treg balance in T cells during sepsis, thereby alleviating lung [32] and intestinal inflammation [43]. Second, this effect might be related to the VDR and its genetic polymorphisms. Studies have revealed that 1,25(OH)_2_D affects target tissues by binding to nuclear VDR, which is expressed in lymphocytes, monocytes, macrophages, and dendritic cells [44]. A prospective study revealed that the Fokl polymorphism of the VDR gene influences immune cell function in sepsis, particularly the CC genotype, which is linked to a reduced mortality rate in ICU patients [45]. Ahmad et al. [46] found that the serum levels of vitamin D-binding protein (VDBP), 25(OH)D_3_, and 1,25(OH)_2_D_3_ were markedly lower in sepsis patients than in healthy individuals. Similarly, a decrease in serum VDBP levels was observed five days after cecal ligation and puncture in mice, supporting a potential a protective role of VDBP in sepsis-induced liver injury. In addition to these immune- and receptor-related mechanisms, vitamin D supplementation might also protect the body by regulating stress and inhibiting cell death. Studies have shown that vitamin D alleviates sepsis-induced acute lung injury by increasing miR-149-5p expression and reducing endoplasmic reticulum stress [47]. Vitamin D also provides neuroprotective effects by inhibiting exogenous histone-induced apoptosis and ferroptosis [48].

This study was based on the large, well-standardized MIMIC-IV database, which includes a substantial sample size, high-quality data, and comprehensive records covering demographics, vital signs, laboratory examinations, medication regimens, disease diagnoses, and outcomes. With standardized and traceable data acquisition, the database offers robust support for real-world studies, thereby enhancing the clinical representativeness and persuasiveness of our findings. Despite these strengths, several limitations of the present study should be acknowledged. First, vitamin D is not a routine test in critically ill patients; therefore, the MIMIC-IV database contains limited relevant measurement data, and the incomplete data may have affected the robustness of the results to some extent. Second, after PSM, some patients with sepsis who received vitamin D therapy lacked complete records of the administration regimen. Therefore, these patients were excluded from the analysis of the association between administration regimens and 28-day ICU mortality, which reduced the sample size and may have introduced some bias into the stability of the results. Third, MIMIC-IV is a single-center retrospective database, and all study participants were derived from a single center; therefore, caution is warranted when generalizing the findings to other regions or different populations, and further validation is needed in specific settings. Fourth, as a retrospective database, MIMIC-IV cannot capture all potential confounding factors, such as the specific reasons why physicians chose to administer vitamin D, the actual timing of administration, and the status of nutritional support; thus, unmeasured confounding may exist. Furthermore, even though we employed PSM to reduce confounding bias, treatment allocation remained inherently non-randomized; therefore, residual confounding and indication bias cannot be completely eliminated. Based on the above limitations, the associations observed in this study still require causal confirmation through prospective randomized controlled trials. Fifth, the database primarily records in-hospital and short-term outcome data and lacks long-term follow-up outcomes after vitamin D intervention, such as long-term mortality and organ function recovery, making it difficult to comprehensively evaluate its long-term benefits.

## 5. Conclusions

In conclusion, this retrospective cohort study suggests that vitamin D3 supplementation is associated with lower 28-day ICU mortality and better outcomes in patients with sepsis. Further large-scale prospective randomized controlled trials are warranted to validate these observational associations and establish causal effects.

## Figures and Tables

**Table 1:** Characteristics of the study population before and after propensity score matching.

Variable	Before PSM					After PSM				
	Total (*n* = 28,524)	No Vitamin D3 (*n* = 26,096)	Vitamin D3 (*n* = 2428)	*p* Value	SMD	Total (*n* = 4856)	No (*n* = 2428)	Yes (*n* = 2428)	*p* Value	SMD
Age, M (Q_1_, Q_3_)	68.0 (57.0, 79.0)	68.0 (57.0, 79.0)	71.0 (62.0, 81.0)	<0.001	0.249	72.0 (62.0, 82.0)	72.0 (62.0, 82.0)	71.0 (62.0, 81.0)	0.191	−0.035
Gender, *n* (%)				<0.001					0.818	
Female	16,571 (58.1)	15,395 (59.0)	1176 (48.4)		−0.211	2344 (48.3)	1168 (48.1)	1176 (48.4)		0.007
Male	11,953 (41.9)	10,701 (41.0)	1252 (51.6)		0.211	2512 (51.7)	1260 (51.9)	1252 (51.6)		−0.007
Weight, M (Q_1_, Q_3_)	80.0 (66.9, 95.0)	80.0 (67.0, 95.0)	77.5 (64.7, 93.4)	<0.001	−0.099	77.8 (65.0, 92.8)	77.9 (65.0, 92.1)	77.5 (64.7, 93.4)	0.659	−0.014
Vitalhr, M (Q_1_, Q_3_)	85.2 (75.4, 97.1)	85.3 (75.5, 97.2)	84.1 (74.3, 96.0)	<0.001	−0.08	84.0 (74.1, 96.0)	83.7 (74.0, 96.0)	84.1 (74.3, 96.0)	0.761	0.003
Labwbc, M (Q_1_, Q_3_)	11.8 (8.5, 15.7)	11.8 (8.5, 15.8)	11.0 (7.9, 15.2)	<0.001	−0.038	11.3 (7.9, 15.3)	11.5 (7.9, 15.4)	11.0 (7.9, 15.2)	0.267	0.036
Labrbc, M (Q_1_, Q_3_)	3.4 (3.0, 3.9)	3.5 (3.0, 3.9)	3.4 (2.9, 3.8)	<0.001	−0.147	3.3 (2.9, 3.8)	3.3 (2.9, 3.8)	3.4 (2.9, 3.8)	0.984	0.004
Labhemoglobin, M (Q_1_, Q_3_)	10.3 (9.0, 11.7)	10.3 (9.0, 11.8)	9.9 (8.6, 11.4)	<0.001	−0.189	9.9 (8.7, 11.3)	9.9 (8.7, 11.3)	9.9 (8.6, 11.4)	0.801	−0.001
Labrdw, M (Q_1_, Q_3_)	14.7 (13.6, 16.4)	14.7 (13.6, 16.3)	15.2 (14.0, 17.0)	<0.001	0.189	15.2 (13.9, 17.0)	15.1 (13.9, 17.1)	15.2 (14.0, 17.0)	0.162	0.001
Labsodium, M (Q_1_, Q_3_)	138.5 (136.0, 141.0)	138.5 (136.0, 141.0)	138.4 (135.3, 141.0)	0.039	−0.049	138.4 (135.5, 141.0)	138.4 (135.5, 141.0)	138.4 (135.3, 141.0)	0.973	0.009
Labpotassium, M (Q_1_, Q_3_)	4.2 (3.8, 4.6)	4.2 (3.8, 4.6)	4.1 (3.8, 4.6)	0.21	−0.003	4.2 (3.8, 4.6)	4.2 (3.8, 4.6)	4.1 (3.8, 4.6)	0.978	0.004
Labcalciumtotal, M (Q_1_, Q_3_)	8.3 (7.8, 8.7)	8.3 (7.8, 8.7)	8.3 (7.9, 8.8)	<0.001	0.092	8.3 (7.9, 8.8)	8.3 (7.9, 8.8)	8.3 (7.9, 8.8)	0.372	−0.004
Labchloride, M (Q_1_, Q_3_)	104.8 (100.7, 108.3)	105.0 (101.0, 108.3)	103.5 (99.0, 107.3)	<0.001	−0.192	103.6 (99.3, 107.5)	103.7 (99.5, 107.5)	103.5 (99.0, 107.3)	0.429	−0.006
Labglucose, M (Q_1_, Q_3_)	129.5 (108.5, 161.0)	129.5 (108.5, 160.5)	130.1 (107.0, 168.5)	0.291	0.043	130.9 (108.0, 166.5)	131.0 (109.0, 165.0)	130.1 (107.0, 168.5)	0.635	0.007
Lablactate, M (Q_1_, Q_3_)	2.2 (1.5, 2.3)	2.2 (1.5, 2.4)	2.1 (1.4, 2.3)	<0.001	−0.096	2.2 (1.4, 2.3)	2.2 (1.4, 2.3)	2.1 (1.4, 2.3)	0.264	−0.005
Labpt, M (Q_1_, Q_3_)	14.8 (13.1, 16.7)	14.8 (13.1, 16.7)	15.2 (13.1, 17.0)	0.001	0.071	15.1 (13.1, 17.1)	15.0 (13.1, 17.1)	15.2 (13.1, 17.0)	0.802	0.005
Labinr, M (Q_1_, Q_3_)	1.4 (1.2, 1.5)	1.3 (1.2, 1.5)	1.4 (1.2, 1.6)	<0.001	0.07	1.4 (1.2, 1.6)	1.4 (1.2, 1.6)	1.4 (1.2, 1.6)	0.516	−0.001
Labcreatinine, M (Q_1_, Q_3_)	1.1 (0.8, 1.7)	1.1 (0.8, 1.6)	1.2 (0.8, 1.8)	<0.001	0.075	1.1 (0.8, 1.8)	1.1 (0.8, 1.8)	1.2 (0.8, 1.8)	0.094	0.004
SOFA, M (Q_1_, Q_3_)	5.0 (3.0, 8.0)	5.0 (3.0, 8.0)	5.0 (3.0, 8.0)	0.877	−0.028	5.0 (3.0, 8.0)	5.0 (3.0, 8.0)	5.0 (3.0, 8.0)	0.696	−0.025
Diaht, *n* (%)				<0.001					0.355	
No	16,937 (59.4)	15,374 (58.9)	1563 (64.4)		0.114	3095 (63.7)	1532 (63.1)	1563 (64.4)		0.027
Yes	11,587 (40.6)	10,722 (41.1)	865 (35.6)		−0.114	1761 (36.3)	896 (36.9)	865 (35.6)		−0.027
Diadm2, *n* (%)				<0.001					0.693	
No	20,064 (70.3)	18,463 (70.8)	1601 (65.9)		−0.102	3215 (66.2)	1614 (66.5)	1601(65.9)		−0.011
Yes	8460 (29.7)	7633 (29.3)	827 (34.1)		0.102	1641 (33.8)	814 (33.5)	827(34.1)		0.011
Diamt, *n* (%)				0.141					0.501	
No	23,822 (83.5)	21,820 (83.6)	2002 (82.5)		−0.03	3986 (82.1)	1984 (81.7)	2002(82.5)		0.019
Yes	4702 (16.5)	4276 (16.4)	426 (17.6)		0.03	870 (17.9)	444 (18.3)	426(17.6)		−0.019
Diacopd, *n* (%)				<0.001					1	
No	26,005 (91.2)	23,865 (91.5)	2140 (88.1)		−0.102	4280 (88.1)	2140 (88.1)	2140(88.1)		0
Yes	2519 (8.8)	2231 (8.6)	288 (11.9)		0.102	576 (11.9)	288 (11.9)	288(11.9)		0

**Table 2:** Outcomes of the study population before and after propensity score matching.

Variable	Before PSM				After PSM			
	Total (*n* = 28,524)	No Vitamin D3 (*n* = 26,096)	Vitamin D3 (*n* = 2428)	*p* Value	Total (*n* = 4856)	No Vitamin D3 (*n* = 2428)	Vitamin D3 (*n* = 2428)	*p* Value
Death within ICU 28 days, *n* (%)				<0.001				<0.001
No	22,568 (79.1)	20,563 (78.8)	2005 (82.6)		3835 (79.0)	1830 (75.4)	2005 (82.6)	
Yes	5956 (20.9)	5533 (21.2)	423 (17.4)		1021 (21.0)	598 (24.6)	423 (17.4)	
Hospday, M (Q_1_, Q_3_)	9.6 (5.8, 17.0)	9.4 (5.7, 16.8)	11.0 (6.3, 20.2)	<0.001	10.2 (6.0, 18.1)	9.7 (5.9, 16.2)	11.0 (6.3, 20.2)	<0.001
Icuday, M (Q_1_, Q_3_)	3.3 (1.9, 6.8)	3.3 (1.9, 6.8)	3.2 (1.9, 6.9)	0.963	3.3 (1.9, 6.7)	3.4 (2.0, 6.6)	3.2 (1.9, 6.9)	0.468
Ventilation, *n* (%)				<0.001				<0.001
No	3589 (12.6)	3204 (12.3)	385 (15.9)		683 (14.1)	298 (12.3)	385 (15.9)	
Yes	24,935 (87.4)	22,892 (87.7)	2043 (84.1)		4173 (85.9)	2130 (87.7)	2043 (84.1)	
Ventilation hour, M (Q_1_, Q_3_)	40.9 (16.1, 96.0)	41.0 (16.8, 96.0)	37.0 (11.6, 92.3)	0.002	39.8 (14.3, 93.2)	42.0 (17.3, 93.8)	37.0 (11.6, 92.3)	0.012

**Table 3:** Cox proportional hazards models for 28-day ICU mortality.

Type	Unadjusted HR (95% CI)	*p* Value	Adjusted HR HR (95% CI)	*p* Value
Before PSM				
No vitamin D3	1.00 (Reference)		1.00 (Reference)	
Vitamin D3	0.79 (0.72, 0.87)	<0.001	0.71 (0.64, 0.78)	<0.001
After PSM				
No vitamin D3	1.00 (Reference)		1.00 (Reference)	
Vitamin D3	0.66 (0.59, 0.75)	<0.001	0.66 (0.58, 0.75)	<0.001

**Table 4:** Hazard ratios for 28-day ICU mortality according to vitamin D3 administration regimens in critically ill patients.

Subgroup	No. of Patients (ICU Deaths)	Unadjusted HR (95% CI)	*p* Value	Adjusted HR (95% CI)	*p* Value
Cumulative dose					
Low cumulative dose	1825 (361)	1.00 (Reference)		1.00 (Reference)	
High cumulative dose	235 (21)	0.41 (0.27, 0.64)	<0.001	0.37 (0.24, 0.58)	<0.001
Daily dose					
Low daily dose	1999 (373)	1.00 (Reference)		1.00 (Reference)	
High daily dose	61 (9)	0.77 (0.40, 1.49)	0.441	0.68 (0.35, 1.32)	0.257
Dosing frequency					
Infrequent	2000 (373)	1.00 (Reference)		1.00 (Reference)	
Frequent	60 (9)	0.80 (0.41, 1.56)	0.516	0.77 (0.40, 1.50)	0.444

## Data Availability

The original contributions presented in this study are included in the article/Supplementary Materials. Further inquiries can be directed to the corresponding authors.

## Supplementary Materials

The following supporting information can be downloaded at: https://www.mdpi.com/article/10.3390/pathogens15040433/s1. Figure S1: Distribution of propensity scores before and after 1:1 PSM in the study cohort; Figure S2: Covariate balance before and after propensity score matching, assessed by absolute SMDs.

## Abbreviations

MIMIC-IV	Medical Information Mart for Intensive Care-IV
ICU	intensive care unit
PSM	propensity score matching
COPD	chronic obstructive pulmonary disease
HR	heart rate
WBC	white blood cell
RBC	red blood cell
RDW	red cell distribution width
PT	prothrombin time
INR	international normalized ratio
