# Supplementary material for: Impact of Vitamin D3 Supplementation on 28-Day ICU Mortality in Sepsis Patients: A Retrospective Study with Propensity Score Matching
Source: Pathogens. 2026 Apr 16;15(4):433. doi: 10.3390/pathogens15040433 (PMC13119377; doi:10.3390/pathogens15040433)
Supplement: Supplementary file 1 [file pathogens-15-00433-s001.zip › pathogens-4143038-supplementary.pdf]

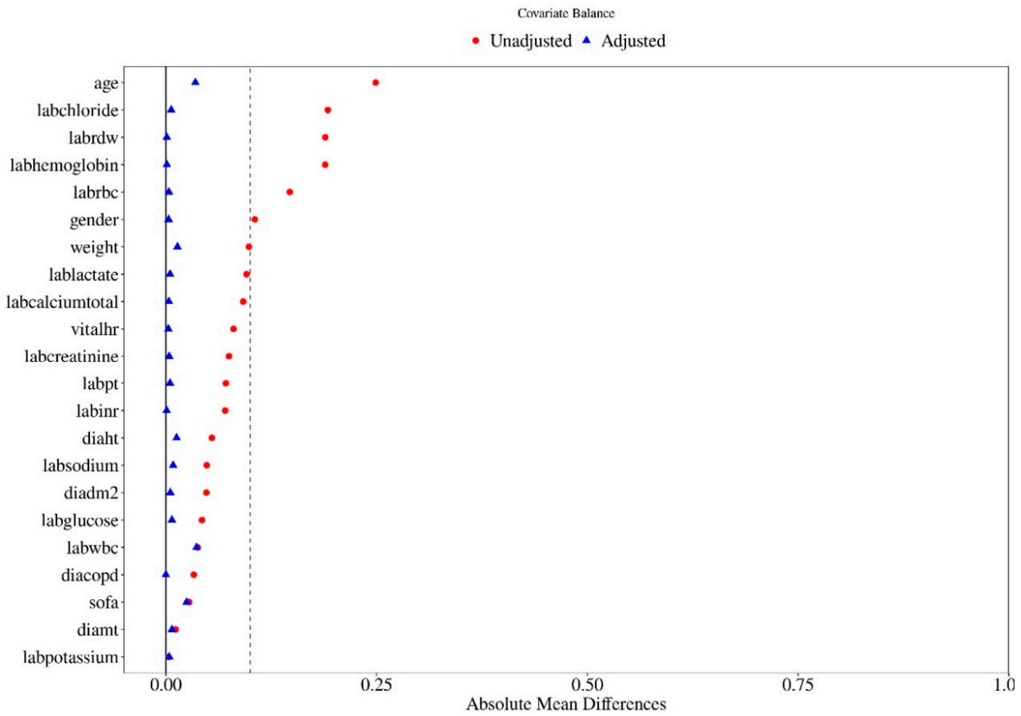

Supplemental Figure S1. Distribution of propensity scores before and after 1:1 PSM in the study cohort.

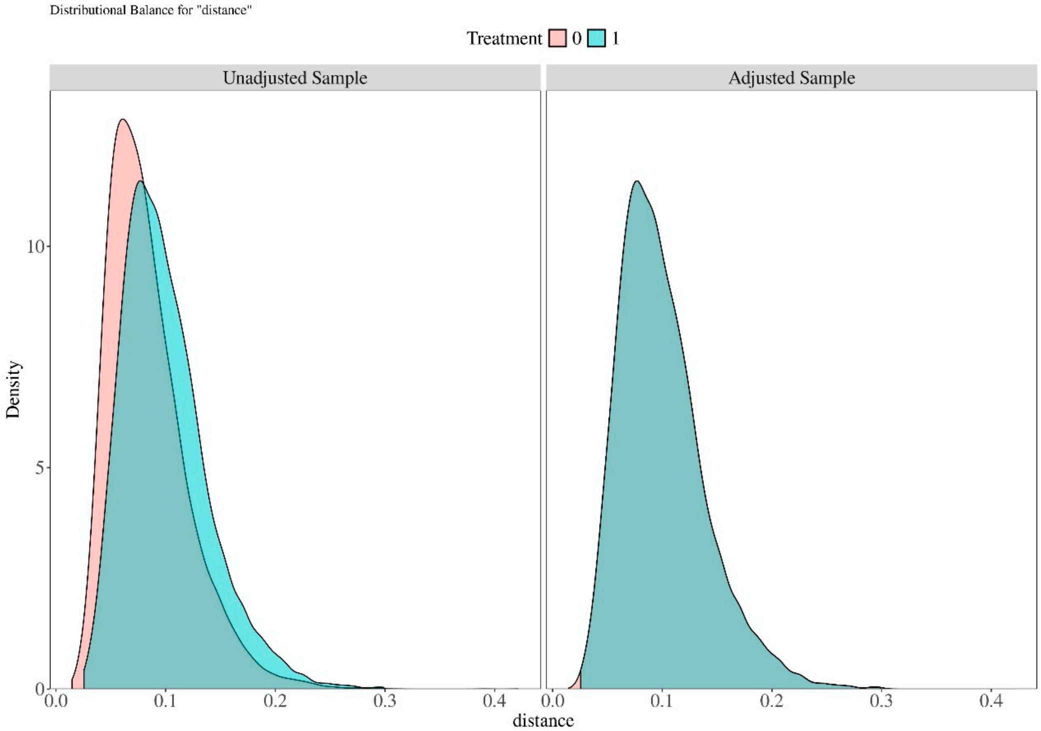

Supplemental Figure S2. Covariate balance before and after propensity score matching, assessed by absolute SMDs.
